# Gut Microbiota: A Key Regulator in the Effects of Environmental Hazards on Modulates Insulin Resistance

**DOI:** 10.3389/fcimb.2021.800432

**Published:** 2022-01-17

**Authors:** Ruixue Huang

**Affiliations:** Department of Occupational and Environmental Health, Xiangya School of Public Health, Central South University, Changsha, China

**Keywords:** environmental, hazards, risk, gut, microbiota, insulin

## Abstract

Insulin resistance is a hallmark of Alzheimer’s disease (AD), type II diabetes (T2D), and Parkinson’s disease (PD). Emerging evidence indicates that these disorders are typically characterized by alterations in the gut microbiota composition, diversity, and their metabolites. Currently, it is understood that environmental hazards including ionizing radiation, toxic heavy metals, pesticides, particle matter, and polycyclic aromatic hydrocarbons are capable of interacting with gut microbiota and have a non-beneficial health effect. Based on the current study, we propose the hypothesis of “gut microenvironment baseline drift”. According to this “baseline drift” theory, gut microbiota is a temporarily combined cluster of species sharing the same environmental stresses for a short period, which would change quickly under the influence of different environmental factors. This indicates that the microbial species in the gut do not have a long-term relationship; any split, division, or recombination may occur in different environments. Nonetheless, the “baseline drift” theory considers the critical role of the response of the whole gut microbiome. Undoubtedly, this hypothesis implies that the gut microbiota response is not merely a “cross junction” switch; in contrast, the human health or disease is a result of a rich palette of gut-microbiota-driven multiple-pathway responses. In summary, environmental factors, including hazardous and normal factors, are critical to the biological impact of the gut microbiota responses and the dual effect of the gut microbiota on the regulation of biological functions. Novel appreciation of the role of gut microbiota and environmental hazards in the insulin resistance would shed new light on insulin resistance and also promote the development of new research direction and new overcoming strategies for patients.

## Introduction

**Figure 1:**
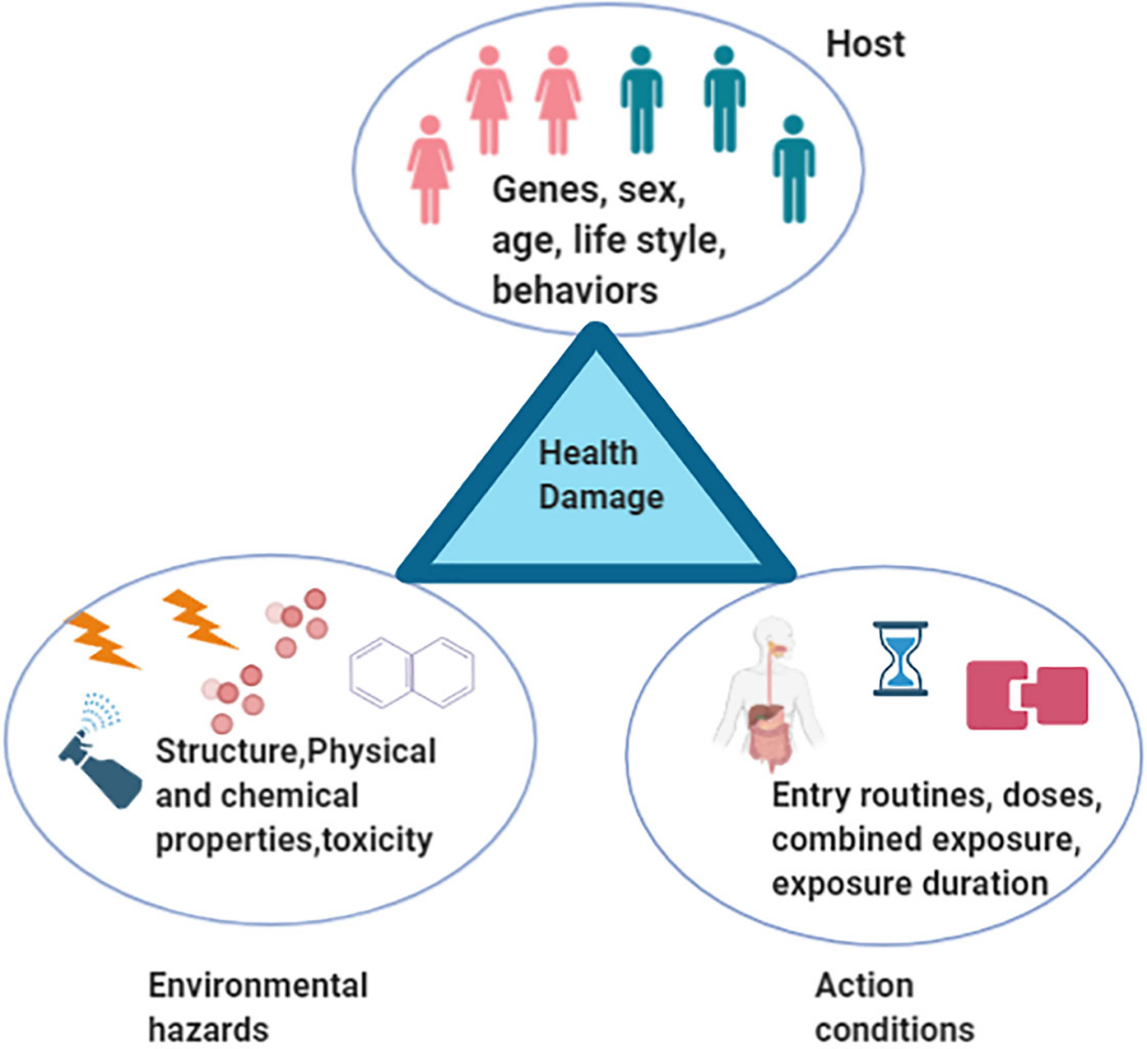
A triangle model that environmental hazards, host, and regulation conditions are three main factors that construct the causative chain to induce health damage.

Insulin resistance, a medical status in which the insulin production is sufficient but the body is failing to make use of insulin properly, is a hallmark of Alzheimer’s disease (AD), type II diabetes (T2D), and Parkinson’s disease (PD) (Cardoso and Moreira, 2020; Xi and Xu, 2021). Recently, a potential causative association between environmental hazards and insulin resistance, and the molecular mechanism underlying the disruption of the insulin signaling pathway have been reported (Bajaj et al., 2015; Zwierello et al., 2020). Environmental hazards include all kinds of environmental agents that adversely influence health or disrupt the ecological balance important for health, safety, and wellbeing (Bradley, 2017; Kosinska and Nitsch-Osuch, 2020). They are mainly classified as physical, chemical, and biological materials. Examples include ionizing radiation, toxic heavy metal, pesticide, particulate matter (PM), polycyclic aromatic hydrocarbons (PAHs), phthalate acid esters (PAEs), and biological factors, including some viruses and bacteria. The respiratory and digestive tracts and skin are the main routes of entry into the human body (DeLeo et al., 2020; Dedrick et al., 2020). Increasing evidence has shown that the effects of environmental hazards on health are influenced by many factors, such as physiological and chemical characteristics, doses, exposure duration, entry routes, combined exposure, human genetic polymorphisms, gender, behavior, lifestyle, and environmental factors. In general, environmental hazards, host, and regulation conditions are the three main factors that construct the causative chain to induce health damage (**Figure 1**). In this review, we focus on the following outline: (i) gut microbiota rides the winds (environmental hazards); (ii) gut microbiota breaks the waves (insulin resistance); and (iii) the gut microbiota potential is harnessed (Dedrick et al., 2020). Based on the current study, we propose the hypothesis of “gut microenvironment baseline drift”. According to this “baseline drift” theory, gut microbiota is a temporarily combined cluster of species sharing the same environmental stresses for a short period, which would change quickly under the influence of different environmental factors. This indicates that the microbial species in the gut do not have a long-term relationship; any split, division, or recombination may occur in different environments. Our review would provide a new insight on the role of gut microbiota and environmental hazards in insulin resistance. Importantly, our ever-increasing appreciation of the interaction of gut microbiota and environmental hazards will hopefully set the stage for more prevention medicine and clinical advances.

## The Interaction of Gut Microbiota and Environmental Hazards

Since the Human Microbiome Project was launched in 2008, our understanding of the effects of microorganisms on health has significantly progressed over the past decade (Gevers et al., 2012). Mechanisms leading to gut microbiota dysbiosis associated with brain diseases could be stimulus dependent. For instance, harmful bacterial overgrowth changes intestinal permeability and increased the access of bacteria or bacterial-induced metabolites from the intestine to the peripheral blood or hepatic portal vein to further activate inflammatory cytokines in enteric neurons (Mulak and Bonaz, 2015). Cells of the innate immune compartment have also been shown to be involved (Powell et al., 2017). The best-understood function of the gut microbiota is its capacity to regulate the brain–gut axis (He et al., 2020; Kaur et al., 2020). In other words, partly due to gut microbiota-induced diseases, such as irritable bowel syndrome (IBS), patients often experience abnormal physiological behaviors (Lee and Tack, 2010; Salonen et al., 2010). Studies on the roles of the gut microbiota in metabolism-associated diseases, such as obesity and diabetes, have also emerged. However, most studies have focused on the metabolism-related inflammation regulation of the gut microbiota (Li et al., 2020) or gut microbiota-associated metabolites, such as tryptophan (Lanis et al., 2017). These studies proposed that the gut microbiota can fuel metabolism-related inflammation (Kim et al., 2016) and that some key gut microbiota-produced metabolites, such as tryptophan, can be involved in metabolism-related signaling pathways, thus affecting the progress of metabolism-related diseases (Gershon, 2013). In these mechanistic studies, the gut microbiota was induced by different environmental stresses, emphasizing that the alteration in gut microenvironment increases gut permeability, leading to alterations in gut barrier function and allowing the bacteria or their metabolites to enter the bloodstream more easily or more difficult. Hence, setting the environmental factors is essential in similar studies. Why the gut microbiota promotes beneficial functions in some organs but is non-beneficial, or even extremely inhibitory or harmful in others, should be elucidated.

**Figure 2:**
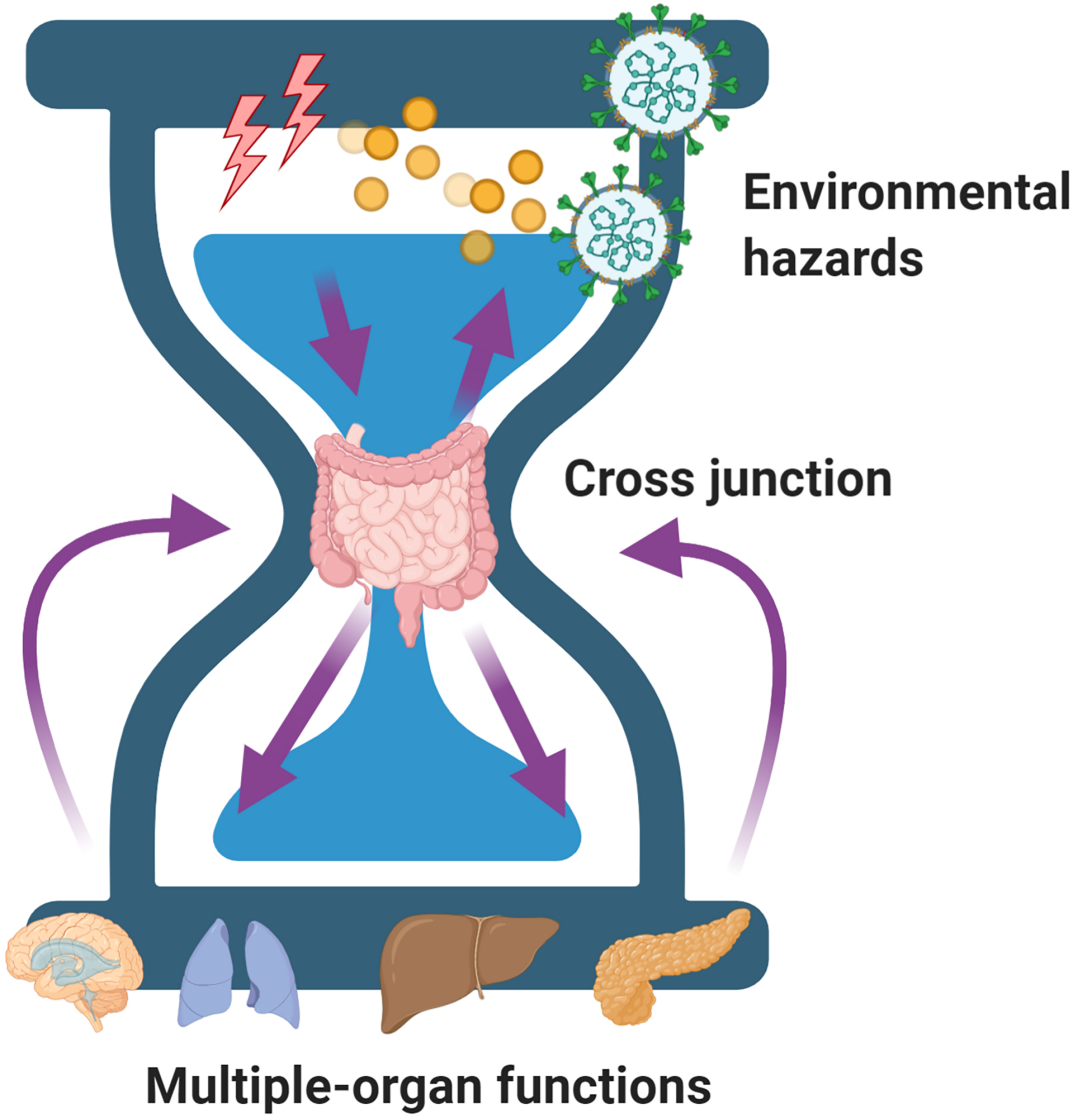
A model of the gut microbiota acting as the “cross junction” of an hourglass that is not only regulated by the environmental factors but also regulates the beneficial or deleterious consequences of organ function.

The setting in which gut microbiota studies could be conducted to provide beneficial or non-beneficial roles have directed our continued thinking as to why gut microbiota is sensitive and how it works as plasticity regulator. On the one hand, gut physiological properties maybe altered by hazardous environmental factors, such as alcohol or ionizing radiation (Huang et al., 2020). On the other hand, it may have the ability to regulate its function to adapt to environmental insults for improved survival by altering its composition or increasing the number of mutations in its genome (Liu et al., 2019). In both hypotheses, the gut microbiota only acts as the “cross junction” of an hourglass that is not only regulated by the environmental factors but also regulates the beneficial or deleterious consequences of organ function (**Figure 2**).

Based on the discussion above on the evolutionary selection, the gut microenvironment affected by the external environment would contribute to altering the gut microbiota, which would thereby perturb the gut microbiota composition or diversity in the human body (Ding et al., 2019). The modified gut microbiota is typically different from the previous composition and leads the gut microenvironment to better adapt to the environmental changes and promote human body progression. However, if we look into the studies evaluating the association among the environment, gut microbiota, and insulin resistance interactions, then the gut microbiota is deemed to be influenced by environmental hazards. In this review, we propose the hypothesis of “gut microenvironment baseline drift.” This hypothesis would challenge the classical consideration of the effects of environmental hazards on health and diseases. Previous studies tend to define the gut microenvironment as integrated. However, according to the “baseline drift” theory, gut microbiota is a temporarily combined cluster of species sharing the same environmental stresses for a short period, which would change quickly under the influence of different environmental factors. This indicates that the microbial species in the gut do not have a long-term relationship; any split, division, or recombination may occur in different environments. Nonetheless, the “baseline drift” theory considers the critical role of the response of the whole gut microbiome. Undoubtedly, this hypothesis implies that the gut microbiota response is not merely a “cross junction” switch; in contrast, the human health or disease is a result of a rich palette of gut-microbiota-driven multiple-pathway responses. In summary, environmental factors, including hazardous and normal factors, are critical to the biological impact of the gut microbiota responses, and the dual effect of the gut microbiota on the regulation of biological functions.

### Physical Factor of Ionizing Radiation

Unlike the direct effect of the oral dietary intake on the gut microbiota (Conteh and Huang, 2020), ionizing radiation affects the gut microbiota approximately through indirect regulation, and the gut microbiota responses to ionizing radiation depend on the radiation type, dose, and contact duration. Environmental ionizing radiation exposure often exists in radiotherapy in cancer patients (Zhang et al., 2019), health examinations using X-rays, or public health emergencies such as the Chernobyl incident (Huang et al., 2020), or space activities (Cervantes and Hong, 2016). Maryam et al. pointed out that remarkable alterations in the microbiome in the feces are attributed to 5–12 Gy radiation exposure with significantly increased abundance of Lactobacillaceae and Staphylococcaceae families and decreased abundance of Lachnospiraceae, Ruminococcaceae, and Clostridiaceae families (Goudarzi et al., 2016).

### Chemical Factors

Many toxic environmental metals, including lead, cadmium, and mercury, have also been reported to be involved in regulating the gut microbiota. Gao et al. indicated that lead exposure increases the abundance of Clostridiaceae but decreases that of *Blautia*, *Coprococcus*, and *Ruminococcus* (Zhang et al., 2020). Mercury, either inorganic or organic, is a globally well-known environmental pollutant. Mice administered 160 mg/L of mercury showed an increase in the abundance of Clostridiales, *Lactobacillus*, *Treponema*, *Oscillospira*, and *Desulfovibrio* but decrease in that of S24-7, *Acinetobacter*, and *Staphylococcus* (Zhao et al., 2020). However, in this study, the authors did not suggest that alterations in the gut microbiota caused by exposure to mercury are associated with oxidative stress biomarkers; this is suggestive of the possibility of existence of other molecular mechanisms underlying mercury-induced gut microbiota alteration. Senait et al. summarized the relationship between toxic metals and intestinal microbiome perturbation and found that metal exposure can alter the composition and diversity of the gut microbiota. Moreover, the gut microbiota may alter the intestinal permeability, bioavailability, and toxicity, eventually mitigating or exacerbating the toxic metal toxicity (Assefa and Kohler, 2020). Although evidence has shown that a profound effect of the toxicity of metals is associated with the gut microbiota, most studies have focused on animal or *in vitro* studies and population-based studies, in particular, a population with occupational toxic metal exposure. Therefore, the functions of the gut microbiota shaped by toxic metals should be evaluated further.

Globally, the application of pesticides has been demonstrated to be significantly associated with a wider range of diseases including systemic ADs, lung damage, and liver diseases. Neonicotinoid insecticide imidacloprid (IMI), one of the pesticides, can disturb the gut barrier and increase the Gram-negative bacteria overload in gut microbiota, resulting in an imbalance of gut microbiota in male C57BL/6J mice (Yang et al., 2020). Zeng et al. indicated that the gut microbiota plays a role in degradation of pesticides (Yang et al., 2020) and in metabolism of pesticides, leading to altered bioavailability, bioactivity, and toxicity (Nakov and Velikova, 2020). Atrazine, another widely used pesticide, is metabolized by rare gut bacteria *Serratiamarcescens* and *Pseudomonas protegens*, causing functional and inherited changes in the gut microbiota (Wang et al., 2020). Thus, from the perspective of Meng et al., the gut microbiota is a key factor in the host health effects induced by pesticides (Meng et al., 2020). Similarly, Chiu et al. reviewed the impact of environmental chemicals on gut microbiota and suggested that pesticide exploration contributes to alterations in the composition, gene expression, function, and health effects in the host, including metabolism, immunity, and neurological function (Chiu et al., 2020). These studies reinforce the notion that the gut microbiota response has the property of baseline drift.

Increased exposure to air pollutants independently leads to various diseases, including diabetes, asthma, and obesity (Bailey et al., 2020). Epidemiological, cellular, and animal studies have provided increasing evidence that PM, such as PM_2.5_ and PM_10_, can alter the gut microbiota and thereby increase the risk of health damage (Alderete et al., 2018). Liu et al. demonstrated that exposure of mice to a PM_2.5_ suspension through intratracheal instillation contributed to the increase in the Simpson index of gut microbiota and an increased ratio of the phyla *Proteobacteria*, *Candidatus*, *Saccharibacteria*, and *Fusobacteria* and decreased ratio of *Acidobacteria*, *Gemmatimonadetes*, and *Deferribacteres* in the gut (Liu et al., 2020).

**Figure 3:**
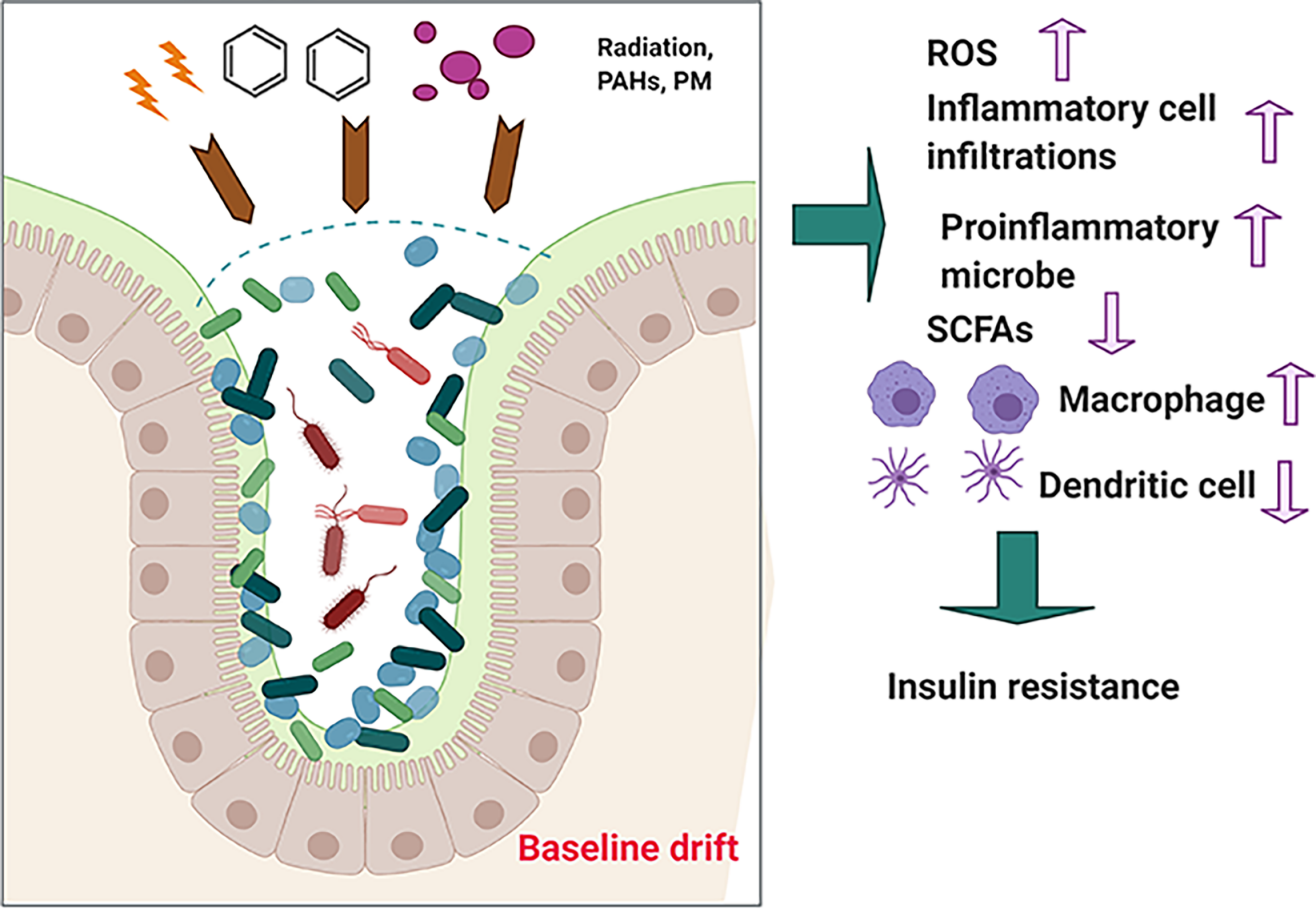
The effects of long-term exposure to low-dose PAH on health.

Consisting of a wide group of chemical compounds with two or multiple fused benzene rings (Manousi and Zachariadis, 2020), polycyclic aromatic hydrocarbons (PAHs) exhibit hydrophobicity and low water solubility. In addition, numerous studies have reported that PAHs are widely spread occupational and environmental contaminants (Leachi et al., 2020). PAHs are mainly byproducts of incomplete combustion of organic materials, including coal, garbage, and gasoline (Pardo et al., 2020). Marja et al. observed that exposure to PAHs from air and soil causes an imbalance in human microbiota, leading to alteration of *Actinobacteria*, *Bacteroidetes*, and *Proteobacteria* communities on children’s skin (Roslund et al., 2019). Gut microbiota can modify the toxicity of environmental PAHs through direct interference or through indirect modulation of the host immune system, indicating that gut microbiota serves as an important modifiable factor for subsequent health influence (**Figure 3**).

Based on our hypothesis that the effects of environmental hazards on the host health may be attributed to “baseline drift” of gut microbiota, we have used some of the above examples to illustrated that “baseline drift” is critical for re-establishing the host health-related gut microbiota communities and that it may be beneficial or non-beneficial for health. From this hypothesis, we can further identify that (i) environmental hazards insult is associated with intestinal gut microbiota dysbiosis; (ii) in response to environmental hazards insult, gut microbiota may alter their composition or diversity to adapt to changes and may produce beneficial products, such as SCFAs, or non-beneficial products, such as proinflammatory cytokines including interleukin (IL)-1β; and (iii) environmental hazard-mediated alteration of gut microbiota may be affected or associated with multiple diseases, such as diabetes and liver damage. Just as advances in our understanding of gut microbiota biology have become complicated instead of simple, the association of gut microbiota with host–environment health has been complicated by the many emerging roles of gut microbiota, in which the alteration of gut microbiota is linked with diseases.

## The Role of Gut Microbiota in Regulation of Insulin Resistance

The gut microbiota has been considered as the human second genome. Alexandre et al. recently retrieved 13,133 human gut meta-genomic datasets to establish a large-scale discovery of uncultured species. This study expanded the appreciation of gut microbiota and highlighted its complicated function (Almeida et al., 2019). Furthermore, the study showed how the gut microbiota evolve for environmental adaptation and how diversity or composition changes are required for evolution.

Insulin resistance refers to the disruption of insulin section of pancreatic β-cells and the deceased sensitivity of organs in glucose utilization (Huang et al., 2019; Huang et al., 2020; Conteh and Huang, 2020). The online Kyoto Encyclopedia of Genes and Genomes (KEGG) database (https://www.kegg.jp/) summarizes that the following pathways may underlie insulin resistance: (i) in muscle cells, *GLUT4* translocation and glucose uptake are inhibited through the PI3K/AKT2/mTOR pathway perturbation; (ii) in liver cells, gluconeogenic genes are increased, whereas glycogen production is reduced by the perturbation of the IRS/GSK-3 pathway; and (iii) there is increased activity of phosphatases, including PTPs, PTEN, and PP2A. Meanwhile, oxidative stress, mitochondrial dysfunction, accumulation of intracellular lipid derivatives, and inflammation also contribute to insulin resistance (Genders et al., 2020; Ryu et al., 2020; Sharma et al., 2020).

Recently, the roles of the gut microbiota and their metabolites in the regulation of insulin resistance have received increasing attention (He and Li, 2020). The interaction, causality, and underlying mechanism have been studied. In 2004, upon the transplantation of the healthy intestinal flora of mice to germ-free mice, the latter showed increased body fat and insulin resistance, indicating the potential relationship between the gut microbiota and insulin resistance (Backhed et al., 2004). However, an increasing amount of evidence has demonstrated that in the crosstalk between the gut microbiota and insulin resistance, the former may exert dual effects, beneficial or non-beneficial, on insulin regulation (Li et al., 2020). The gut microbiota not only produces beneficial metabolites, such as triacylglycerol, that promote energy absorption but also serves as source of endotoxins that trigger inflammation activities, thus resulting in insulin resistance (Cani et al., 2007). Furthermore, some insulin receptor-related signal pathways exhibited effects on the modulation of inflammatory response or the production of gut microbiota-related metabolites (He and Li, 2020).

Although a number of studies are attempting to elucidate the potential mechanisms underlying the crosstalk between the gut microbiota and insulin resistance, many questions remain unanswered. First, are there studies with conflicting outcomes regarding the effects of gut microbiota composition and diversity alteration on insulin resistance? Second, because most studies were performed using a mouse model or *in vitro*, epidemiological cohort studies are lacking. Third, how the changes in the gut microbiota affect the development of insulin resistance remain unknown. Lastly, although environmental influence is an important factor in these studies, most studies have only focused on the contributions of obesity, diet, unhealthy behaviors, and genetic and epigenetic on insulin resistance, rather than on environmental hazards (Kampmann et al., 2019; Najjar and Feresin, 2019). For instance, a recent cross-sectional study revealed that occupational exposure to chronic ionizing radiation increased the incidence of PD (Azizova et al., 2020). Furthermore, diabetes can promote radiotherapy-induced radiation pneumonitis (Kong et al., 2019). Moreover, some studies revealed that before and after cancer treatment by radiotherapy, gut microbiota dysbiosis was associated with late metabolic complications among childhood cancer survivors (Morel et al., 2020). Another study further showed that exposure to air pollution led to endocrine disruption by altering the commensal bacteria (Roslund et al., 2019). In addition, a population-based epidemiological study found that PM2.5 was positively associated with the risks of impaired fasting glucose and type 2 diabetes *via* a partially mediated gut microbiota composition (Liu et al., 2019). Studies on heavy metal exposure have also shown that increased incidence of diabetes is mediated by the gut microbiota (Li et al., 2019). Particularly, cadmium chloride (Cd) exposure significantly changed the mouse gut microbiome and resulted in a significantly lower microbial diversity, whereas sodium arsenite (As) caused a non-significant decrease in microbial diversity, which was significantly associated with metabolic health (Li et al., 2019).

In fact, epidemiological and laboratory experimental results obtained over the past two decades regarding how the gut microbiota shapes insulin resistance have hinted that certain gut microbiota possess “baseline drift” to the “gain-of-function” properties that produce emerging functions distinct from ancient phenotypes. One of the most prominent functions generated by such evolution in the adaptation to environmental hazards is their potential to regulate insulin resistance (Chakaroun et al., 2020; He and Li, 2020; Vetrani et al., 2020). Whereas the proposed insulin resistance regulatory activities have double-edged sword effects, an emerging rule of thumb is that environmental hazards related to insulin-resistance-derived gut microbiota “baseline drift” may be opposed to the gut microbiota function without exposure to environmental hazards. In any case, not all “baseline drifts” functionally affect insulin resistance, further hinting that the selection intensity of environmental hazards varies based on the exposure dosage and duration.

**Figure 4:**
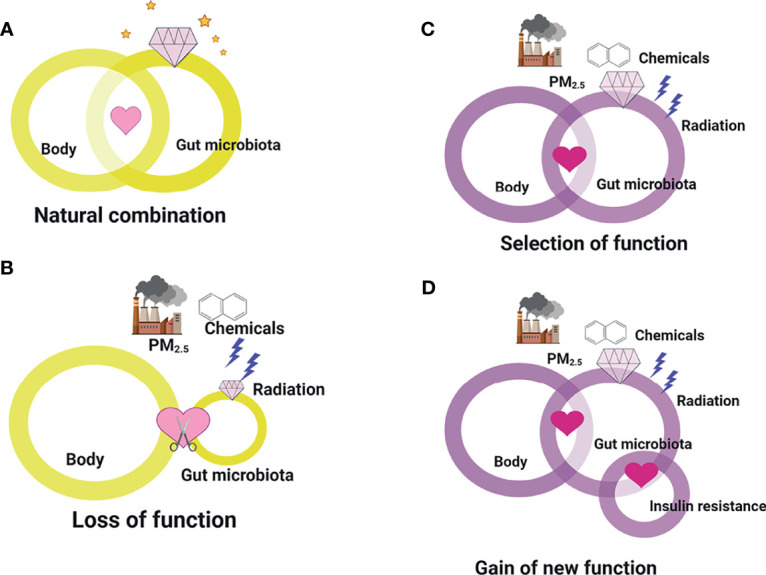
The loss, attenuation, or strength of function, or emerging function of gut microbiota “baseline drift”. **(A)** Under normal conditions without environmental hazards, the human body provides residence for microbiota survival, whereas the gut microbiota produces metabolites to maintain health balance. **(B)** Upon the introduction of environmental hazards, the protective role of the gut microbiota on health may be lost. **(C)** When the dosage and exposure duration are not extensive, the gut microbiota may undergo selection and gain new functions. **(D)** Certain conditions may lead to specific microbiome mutations, yielding new species that may gain new functions, such as the induction or promotion of insulin resistance.

Indeed, a distinct phenotype of the gut microbiota is not sufficient to define a “baseline drift” as “gain-of-function.” Theoretically, gut microbiota “baseline drift” may reflect the loss, attenuation, or strength of function, or emerging function. As illustrated in **Figure 4A**, under normal conditions without environmental hazards, the human body provides residence for microbiota survival, whereas the gut microbiota produces metabolites to maintain health balance. However, upon the introduction of environmental hazards, the protective role of the gut microbiota on health may be lost (**Figure 4B**). It may be damaged during the alteration of its composition and diversity, resulting to a failure in sustaining health. However, when the dosage and exposure duration are not extensive, the gut microbiota may undergo selection and gain new functions. Similar to the gut microbiota, it tended to change its partner to find a newly selected one (**Figure 4C**). In fact, the emergence of new function of the gut microbiota in response to environmental hazards is common, whereby the gut microbiota can retain its regulatory function and interact with critical body organs. Finally, certain conditions may lead to specific microbiome mutations, yielding new species that may gain new functions, such as the induction or promotion of insulin resistance. Although the gut microbiota normally interacts with the human body, the emergence of “baseline drift”-induced function shows how the gut microbiota has the potential to play another function as well (**Figure 4D**). In fact, although the gut microbiota with changed function is generally classified based on its effect on the composition or diversity of metabolites, it is currently impossible to accurately predict how specific microbiome influences regulation. In addition, determining the occurrence of various distinct yet selective and lost environmental hazard–gut microbiota–host interactions is impossible. Collectively, our emerging appreciation of the gut microbiota with changed functions is increasing as we understand how “the most critical function of facilitating the crosstalk between environment and health” promotes or inhibits the development of insulin resistance. Despite the double-edged effects of the gut microbiota in response to environmental hazards, they both contribute to insulin resistance that goes beyond the environment itself. Hence, based on the discussion above, for the clinical application of gut microbiota and clinical decision making in the future for transplantation or intervention targets, the current view on the gut microbiota, i.e., serving simply as “a new organ” or “emerging target,” has to be replaced to consider the potential influences of environmental factors.

## Harnessing the Gut Microbiota Potential

Recently, due to the potential of the gut microbiota in the regulation of health and the alteration of its composition and diversity in response to environmental hazards, strategies targeted for the treatment of environmental-hazard-induced diseases have been developed (Dosoky et al., 2020; Jones and Molloy, 2020; Szydlowska et al., 2020). Among these functions, the potential value of engaging the gut microbiota in response to environmental factors is clear from reported literature, illustrating that robust responses to insulin resistance may depend on the natural gut microbiota composition and diversity alteration, and the potential of metabolites (Luc et al., 2021). For instance, clinical trials have demonstrated that the effects of probiotics on insulin resistance are associated with the reversal of the gut microbiota composition and diversity (Kesika et al., 2019). Although the use of fecal transplantation and probiotics for the therapy of insulin resistance has been attempted, efforts to effectively improve insulin resistance for therapeutic benefit are still underway (Dabke et al., 2019; Zhang et al., 2019).

Here, let us consider fecal transplantation. A clinical, randomized, placebo-controlled study has reported fecal transplantation outcomes; however, they were controversial, as hepatic insulin sensitivity was not observed in both obese patients and healthy controls (Zhang et al., 2019). Although fecal transplantation has potentially higher efficacy rates, there are still many challenges for its clinical use, e.g., how to qualify stool donation, donor inclusion and exclusion criteria, and ethical concerns (Dubois et al., 2020). In the future, studies involving a larger sample population and those elucidating the relative molecular mechanisms should be conducted to improve clinical outcomes and address challenges regarding accessibility, acceptability, lack of standardization, and regulatory complexity of fecal transplantation (Fadda, 2020).

In addition, another targeting strategy to prevent insulin resistance is the use of probiotics (Alard et al., 2016). The administration of probiotics, including *Lactiplantibacillus plantarum*, can improve insulin sensitivity and gut microbiota diversity (Guimaraes et al., 2020; Sharma et al., 2020). Because the ratio between the phyla Firmicutes and Bacteroidetes is particularly important for insulin resistance, probiotics have been shown to have beneficial effects in restoring the gut microbiota composition and maintaining the said ratio (Sharma et al., 2020). The relative mechanisms underlying these therapeutic effects have been found to be associated with downregulated insulin signaling, such as that of the transcription factor FOXO1, and the prevention of methylation and demethylation of H3K79me2 and H3K27me3 (Sharma et al., 2020). In addition, probiotic supplementation can provide beneficial effects on intestinal barrier function and improve inflammation and glucose tolerance (Lee et al., 2019). Nevertheless, some clinical trials showed unfavorable results of probiotic supplementation (Kesika et al., 2019). However, it must be noted that probiotics supplements contain blends. In addition, host dietary habit and lifestyle may also influence their efficacy. Therefore, future studies are necessary to determine the role of each specific probiotic blend in modulating insulin resistance and how host diet and lifestyle affect their activities.

Furthermore, we searched the online International Clinical Trials Registry Platform established by the World Health Organization for human clinical trials using the keywords “FMT” (fecal microbiota transplantation) and “probiotics” (http://www.chictr.org.cn/index.aspx). Compared with the 281 available records for FMT trials, 2,295 probiotics therapy-related trials have been registered aiming to explore its effect on various diseases, including diabetes and obesity. Because the molecular mechanisms underlying the therapeutic effects of FMT and probiotics in insulin resistance remain unknown, registered clinical trials should compare the therapeutic efficacy, whereby the results may be translated to clinically improve insulin resistance.

In theory, insulin resistance has the ability to escape immune editing; thus, there is an increasing interest in combining gut microbiota immunotherapy with insulin resistance. In a mouse study, Bhat et al. found that mice treated with immune suppressants and probiotics reversed the baseline of *Oseburia*, *Oscillospira*, *Mollicutes*, *Rothia*, *Micrococcaceae*, *Actinomycetales*, and *Staphylococcus* abundance and showed a marked increase in sucrose degradation. This suggested that modulating the composition of the gut microbiota using probiotics might improve diabetes *via* immune suppression (Bhat et al., 2017). Vincent et al. demonstrated that insulin resistance in mice fed with high-fat diet was due to an adaptive immune response specific to certain pathogens (Blasco-Baque et al., 2017). Therefore, immunotherapy for the gut microbiota to improve insulin resistance is an attractive approach. Accordingly, strategies combining immunotherapy and other probiotics, FMT, or dietary intervention should be explored in future studies. In addition, while the mechanistic basis of therapeutic efficacy and safety is critical for mining new targets and signaling pathways for intervention, further appreciation of the baseline drift of gut microbiota biology will be required for successful clinical applications.

## Concluding Remark

**Figure 5:**
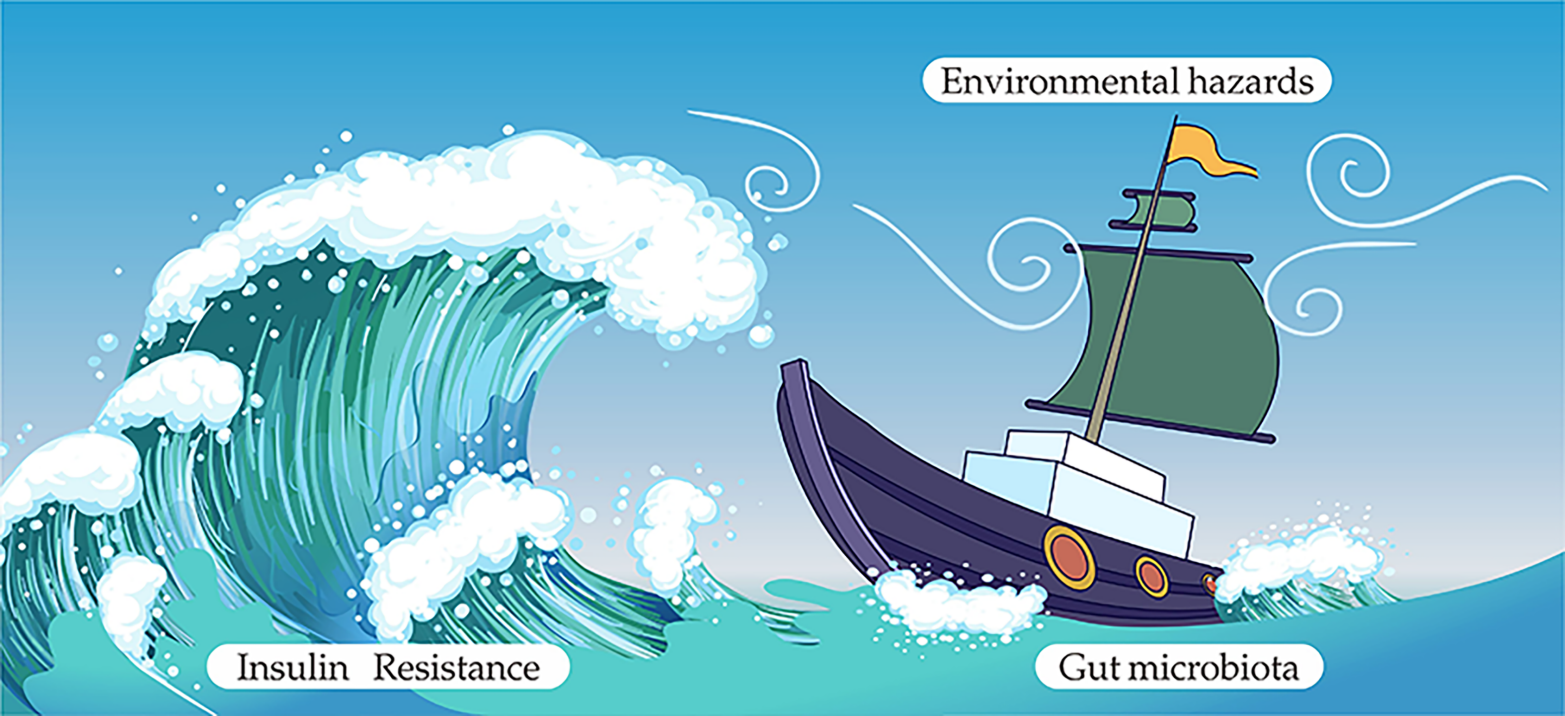
An illustration showing that in facing the challenges of environmental hazards and insulin resistance, the gut microbiota can ride winds (environmental hazards) and break waves (insulin resistance).

Over the past decade, the gut microbiota has captured the attention of biologists, physicians, and the public. With increasing in-depth studies, detailed understanding of the gut microbiota has provided us important insights into the molecular mechanisms underlying insulin-related regulation. Although the findings of some studies on the insulin modulation by the gut microbiota are contradictory, the essential role of environmental hazard exposure in its responses cannot be denied. This results from the complicated interaction between the gut microbiota and host, triggering insulin signaling pathways and mediating insulin-receptor-related systems, such as the liver, muscle, and adipose tissues. While these effects on insulin resistance may depend on the “baseline drift,” understanding how it affects and causes the “baseline drift” in the crosstalk with insulin regulation is a challenge in the next decade. It should be noted that deleterious consequences of gut microbiota on insulin-sensitive and other important organs should be avoided. The following importance and challenges should be addressed urgently. First, how is the “baseline drift” significant in the regulation of insulin-sensitive organs and host? Second, what are the mechanisms involved in shaping the gut microbiota “baseline drift” in response to environmental hazards? Third, how are the effects of multiple environmental hazards combine effects rather than single hazard factor exposure on insulin resistance? Lastly, how should the complicated results of numerous studies be managed for clinical application? Indeed, the difficulties in exploiting the gut microbiota therapeutically may hinder the mitigation of insulin-resistance-related diseases. Without novel therapeutic innovations, insulin resistance will continue to be associated with increasing global morbidity from diabetes or other diseases. The current knowledge in the gut microbiota has only recently emerged, and we are hopeful that future studies would yield compelling and more robust clinical intervention. In the end, we would like to use **Figure 5** to show that gut microbiota is just like a sailing boat on the sea. While facing the challenges of environmental hazards and insulin resistance, the gut microbiota can ride winds (environmental hazards) and break waves (insulin resistance).

## Data Availability Statement

The original contributions presented in the study are included in the article/supplementary material. Further inquiries can be directed to the corresponding author.

## Author Contributions

RH conceived and designed the study, searched and reviewed literatures, drafted the illustrations and initial manuscript, and RH critically reviewed and revised the manuscript.

## Funding

This study was supported by the National Natural Science Foundation of China (Grant Nos. 82073486 and U1803124) and the Natural Science Foundation of Hunan Province (Grant No. 2019JJ40396).

## Conflict of Interest

The author declares that the research was conducted in the absence of any commercial or financial relationships that could be construed as a potential conflict of interest.

## Publisher’s Note

All claims expressed in this article are solely those of the authors and do not necessarily represent those of their affiliated organizations, or those of the publisher, the editors and the reviewers. Any product that may be evaluated in this article, or claim that may be made by its manufacturer, is not guaranteed or endorsed by the publisher.
